# Quack Advertisements in Religious Newspapers

**Published:** 1858-01

**Authors:** 


### Editors' Table.

### Quack Advertisements in Religious Newspapers.

"While thou so hotly disclaimest the devil, be not guilty of diabolism."—Sir Thomas Browne's Christian Morals.

It is painful to believe that there should be editors of religious newspapers who, while upholding the great truths of the gospel, and expatiating on the beauties of uprightness, justice, purity, and disinterestedness, are ready at the same time, by a singular and inexplicable inconsistency, to give currency to falsehood, fraud, hypocrisy, and venality, in the quack advertisements which disfigure and disgrace their columns. Eros and Anteros, topaz and ebony, the good and evil principle, the spiritual and the corrupt, are brought together in forced conjunction, and are thus made to appear as if they were fit company the one for the other. An imaginative person might see in these newspaper practices a type of man's double nature, and a lesson accordingly; but we must earnestly protest against such lessons, as they carry with them a moral taint, and are damaging to man's health—they impart a knowledge of evil, but without that of any good to redeem it. The mendacious compounders, venders, and advertisers of nostrums and panaceas lay bare, in their descriptions, all the infirmities and diseases to which the human body is subject, but in such a style of exaggeration and perversion as to alarm and disgust; and they are most pleased when they produce this effect, as on it depends their success in extracting money from their victims. Between their promises and realization there is an impassable gulf, wider even than between fact and fiction; for, while the latter is a fanciful imitation of the former, their promises are entirely out of the line of fulfilment.

On what grounds do these leech-empirics receive the patronage, for it amounts to this, of many of the clerical brethren who conduct newspapers; and it may as well be said, also, of many more who are not connected with the press, except, indeed, so far as relates to the publication of their certificates in favor of empiricism and imposture? Quacks, on the score of intellect and education, may be divided into two classes: One, and it is the most numerous of the two, consisting of the ignorant and the illiterate, who are too stupid or too lazy to follow any regular course of honest industry; the other, composed of those who have been unsuccessful in honest callings, and who, becoming desperate, take to the great field of quackery, as others have done to the high-road and the high-seas, where they became robbers and pirates. There is this essential difference, however, between the quack and the robber or the pirate; that, while both parties are intent on emptying the purses of those whom they encounter, the former pursues his vocation with the cant of benevolence and disinterestedness in his mouth, and although he may kill his dupes by the administration of his nostrums, he is careful not to expose his own life; whereas the latter frankly avows his purpose, and at the very time that he takes the life of any one who resists him, he exposes his own without hesitation.

It is universally admitted, that, for a person to be able to repair a machine of any description, whether it be a watch or a steam-engine, which has been broken, or otherwise disordered in its movements, he must have been previously acquainted with the several parts of this machine, and the manner in which each of them is connected with, and acts, and is acted on by the other parts. The human body, with its complicated mechanism, offers no exception to this plain common sense axiom; but, on the contrary, in studying it we are required not only to make ourselves acquainted with its mechanism, so far as this can be explained on the principles of physics, but also with the additional and paramount offices of a vital and peculiar kind, which come under the head of physiology, and which cannot be understood without a close observation of the structure of the organs of the body, and of the modifications impressed by all the external causes continually acting on it—such as air, food, etc. Do the quacks, who profess to cure all diseases, or even any one disease in all its stages, bring with them a knowledge of this kind? We are safe in asserting that not one in fifty of their number is acquainted with the mechanism of the heart and the circulation of the blood; and still less with the composition of this vital fluid, which they so generally assert to be the seat of disease, and for the purification of which they boast of their having found the secret. They know not the process by which food is converted into blood, nor those others by which this blood is made to nourish and build up all the organs, nor those again by which it is relieved by the extrication of gaseous and other substances, the retention of which would derange and finally arrest all the movements of the animal economy. How can it be expected or believed that these impudent pretenders, who are ignorant of the constituent parts of the blood, can tell which of these is in excess or deficient; which of them imparts the red color that gives the glow of health; which to nourish the organs; which to pass through them and be discharged as secretions from various emunctories. And yet these persons boldly claim to have a knowledge of means—peculiar and secret remedies discovered by and only known to themselves—by which the blood of the sick can be at once purified and enriched! Stranger still, these cheats find ready advocates and vouchers in the members of the sacred profession, teachers of gospel truth, and sticklers for the right at all hazards! The age of miracles has passed away; but yet our reverend expositors of the biblical records can persuade themselves, and try to persuade others, that men who but yesterday left the workshop, or the factory, or the farm, ignorant, often stupid and illiterate, are able at once, without any preparation or learning, to look into the complicated organization of the human body, to measure all its derangements, and to restore it to its healthy condition by the administration of substances which are either inert, or which have been used for centuries before by regular physicians. Years of patient thought, careful observation, and laborious research are deemed indispensable conditions for the votaries of medical science to gain even a partial insight into the mysteries of disease; and, after all, they feel that the difficulties which obstructed their path are very imperfectly overcome, and that they can do little more than furnish a good standpoint for the further progress of those who come after them. It is not so with the self-sufficient, suddenly-inspired quack, who, like Minerva, sprung from the brain of Jupiter, is in full panoply, ready for every emergency; no problem too abstruse, no mystery too obscure, but that he can solve the one and illuminate the other. The annals of science, literature, and the arts abound in examples of men eminent in these departments, and in the subdivisions of each of them, who had to contend against poverty, the want of early education, and of opportunity; but none of these men ever reached his eminence at a single leap; no one boasted that he was an astronomer, and had discovered new planets, simply by looking for the first time through a telescope, even were it a Herschell's or a Rosse's. Fulton did not apply steam to navigation, nor Morse electro-magnetism to the telegraph, by intuition and simply willing it. Powers and Crawford were not able to extemporize, in the first week of their noviciate, those beautiful productions of the chisel that have given them a world-wide fame. Genius has never yet been able to realize its aspirations, nor to furnish material for the world's history, without time, labor, and strenuous and persistent effort. But all the examples of history, all the teachings from analogy and experience in every-day life, are set at naught; and dullness and ignorance, as embodied in quacks, are, by the asseverations of certain of the clerical brethren, to obtain credence for discoveries beyond that of steam navigation and the telegraph, for exhibition of genius surpassing those of the sculptor and the painter.

Does it not sometimes occur to sincere Christians, that their excessive credulity in giving so ready an assent to quackery and its alleged miracles, for such they are if its pretensions and statements have any foundation in fact, furnishes a strong excuse to the skeptic in religion, who, judging of the unknown from the known, of a religious creed from a medical creed, can hardly persuade himself of the soundness of the first when he knows the palpable absurdity of the second; nor can he have a high opinion of Christian morals when he sees Christian men aiding and abetting the impostures and falsehoods of the makers and venders of quack medicines? Is it not to be feared that the skeptic and the scoffer will, in a spirit of plausible induction and generalization, proclaim these reverend gentlemen, who are such strenuous advocates of medical quackery, to be quacks in theology, and pretenders to piety, and taking them as representatives of the entire order, to assert that they are all either the most credulous of men, and, therefore, of no authority as teachers, or the greatest of hypocrites, and, therefore, no exemplars or guides for conduct? Such conclusions are not, we admit, very logical, but they are strictly in the line drawn by the clerical allies and abettors of medical quackery.

A plausible explanation of the opposition to legitimate medicine manifested in the undisguised support of the bastard branch, by the parties just mentioned, was given some years ago in the pages of our cotemporary, the *London Lancet*, when discussing the subject of "Quacks and their Abettors." It remarks, "We really think that the church uses medicine as a kind of safety-valve for doubt and disbelief; or else that it must feel a latent jealousy against science for overturning the miraculous virtues of saints and relics. Certain it is that, after a manner, Physic and the Church, two of the black Graces, are in a state of open war. Reverend gentlemen who are straight-laced in their religious creeds, seem to find comfort in disbelieving all that the profession have to advance in favor of medical orthodoxy. Free-thinking, skepticism, and total unbelief in regular medicine would seem necessary to the weakness of the clerical mind; and its vagaries are spent upon our unfortunate art instead of upon matters of religious belief. Clerical quackery is but a mild atheism or infidelity." In a graver strain, when speaking of the mischievous consequences of clerical abetting of quackery, this journal says: "In the same way that we have given, from time to time, lists of medical testimonials in favor of quackery, we propose to give a few certificates from divines of different persuasions. A very amusing pastime this would be, if we could forget that while clergymen are indulging in their medical buffooneries, the health, and even the lives of thousands of their fellow-creatures are constantly being sacrificed to the Moloch of quackery."

We look upon quack advertisements in papers edited by clerical personages in precisely the same light as similar advertisements in other vehicles, to the tail of which the certificates of clergymen are appended. The company in both cases is the same, and the fellow-feeling of the two parties is equally conspicuous. Their logic is common property, the possession of which no one out of their own circle will dispute with them. If clergymen covet notoriety thus obtained, we might leave them to the enjoyment of their tastes, were we not, on the score of humanity, called upon to deplore the effects of such perversions.

On taking up a quack's "Almanac" we see in the titles of some of his compounds a show of their adaptation to particular classes of diseases; but the temptation cannot in any instance be resisted of claiming for the compound a curative power over all the diseases of an organ or apparatus, whether they be acute or chronic, nervous or inflammatory, hemorrhagic or tuberculous, or whether located in tissues quite different from each other in their structure and vital properties. An expectorant, for example, is formally announced as the remedy in asthma, not only to excite the mucous membrane of the lungs to relieve itself by expectoration, but also, and with equal confidence, in hemorrhage from the lungs, in which we are desirous of keeping this organ as quiet as possible, and of preventing any excitement and movement such as are desirable to produce expectoration. As might have been anticipated, we find certificates of clergymen in favor of the "expectorant," which is announced as a wonderfully curative agent in asthma, cough, and influenza. We are not told whether subsequent attacks of the first-mentioned disease—the most formidable of the three—were prevented by this nostrum. For the relief of the fit or paroxysm, there are scores of remedies, each one of which might claim certificates of its curative powers over a stage of the disease that would have passed away without the use of any remedy at all. Under such circumstances the one last taken will have the credit of the cure, we ought rather to say of a solution of the fit of asthma.

But commend us to the out and out spoken praises lavished by another reverend, first on the "expectorant," for its having been the means, "under God," of keeping him from his grave; ditto on the "alterative," for his wife; then on the "carminative balsam," for taking him, who laid as cold as a corpse, out of the jaws of cholera. Next are mentioned "the sanative pills," which "work to a charm;" but whether on the bowels or the brain—to carry off redundant bile, or to relieve from thick coming fancies—we are not told. The most wonderful wonder, to use the words of the itinerant showman, yet remains in the shape of the "vermifuge," which is the best thing the reverend brother ever found among children for the cure of—worms? no, nothing of the sort—it was "for chills and fever." This feat of killing an object not aimed at, beats even the engineering skill of the famous Todlieben in Sebastapol; and can only find a parallel in the annals of cockney sporting. The zealous certifier tells his quack friend of his having "had formerly some practice in medicine, but abandoned it to become a ——— preacher." It is devoutly to be hoped that his theology rests on a more orthodox foundation than his pathology, and that his preaching is more successful than his physicing; for otherwise his flock cannot be said to feed on pastures green, nor to be safe against wolves leaping into the fold.

Jealous perhaps of the reputation which might accrue to the —— church by some of its members being such successful media for the introduction and working of the "expectorant," the "carminative balsam," the "sanative pills," and the "vermifuge," out comes a brother, of another sect, whose rapid cure shows his transmissive power of the "alterative," for that was the article employed in his case, to go far beyond that of his reverend rival of the other denomination. We learn from his certificate that he was seized in the night, with what he afterward learned was "malignant erysipelas," from which he suffered terribly the next day; but we must give the description of the case in his own words:

"I had retired to bed about eleven o'clock in good health, and fell asleep, but was soon awakened by extreme pain in one hand and arm. I succeeded in keeping my bed until about four o'clock. When I arose I found my head badly swollen and in a high state of inflammation. A small black spot on the back of my hand, about the size of a five-cent piece, soon rotted and came out to the bone. When I arose the pain was very severe, running into my head, and even the whole system. By twelve o'clock my head and face were badly swollen. The glands of my throat swelled very much, and by this time every tooth in my head was more or less loose; two-thirds of the skin of my mouth and lips peeled off; my sight quite affected; no physician near. I solicited my friends to take me home, (fifteen miles,) but they were fearful I could not stand it, when T. R. Davenport, who kept the public-house, requested me to go to his house. Chills, faintness, and sickness were constantly increasing upon me. I had become almost insensible."

The pathologist who reads this marvelous statement will notice the unparalleled rapidity with which the disease ran its course, in the first twelve hours. In this period, alterations of structure took place which commonly require days; in four hours a spot on the back of the hand had become gangrenous, the soft parts sloughed, and the bone beneath was exposed. We sometimes hear of a galloping consumption; but such steam-power racing inflammation is without precedent. After this the desquamation of the skin of the mouth and lips, in twelve hours, though in itself wonderfully rapid, ceases to surprise. But relief soon came in the shape of the "alterative," administered by a kind old lady who met the patient at the store. The dose was a spoonful (tea or table not specified) "and repeated two or three times in the course of an hour, in which time the faintness, sickness, and chills had principally left, a free perspiration was on the surface, and the inflammation fast abating. The third day after I was able to ride home. Dr. Plimpton informed me that the attack was one of the severest kind of *malignant erysipelas*, and that the use of your alterative was the means of saving my life." The grateful certifier ought to have made the whole story complete, by expressing his belief that he was a subject of so extraordinarily violent a case of malignant disease, in order to show the still more extraordinarily curative powers of the nostrum used by him on the occasion. We would not treat with levity any form of suffering; but we have shrewd suspicions that the editor and proprietor of the quack almanac has been hoaxed by some wag's describing as a case of malignant erysipelas in a ——— preacher, the effects of a "free fight" in which some unfortunate wight had been engaged and was "punished" by a big-fisted antagonist. Of one thing we are sure: that, if such a narrative had been written by a medical man, we should have no hesitation in taxing him with having—most extravagantly drawn on his imagination.

After such a miraculous cure as that related by the last-mentioned brother, the cures of cholera ushered into notice under the inspiring auspices of a —— D.D., who had also been president of a college down East and Secretary of the American and Foreign Bible Society, seem stale and flat. We should have expected better things from this gentleman, and a more discriminating "compassion for suffering humanity" than to allow himself to figure in a quack almanac, as an advocate of quackery, under the evils of which humanity is continually suffering. What would he say of a man professedly benevolent and pure-minded who should give his testimony in favor of Mormon practices? And yet this person would not sin more against Christian morality than the reverend brother does against the real interests of humanity and truth, in the testimony which he thinks he cannot withhold, in favor of quackery. Among the "miscellaneous certificates" in the almanac, is one from a reverend professor of theology, who, after praising the nostrums, lauds the maker and vendor of them, and asserts that Dr. ——, with whom he has the gratification of a personal acquaintance, "sustains a high reputation in Philadelphia as a regular and skillful physician." Now we happen to know that the person (Dr. ——) named sustains no such reputation in either of the particulars specified; and that there is no justifiable ground or even a reasonable excuse for the reverend professor going so far ahead of the facts. This is, however, but a specimen of the unscrupulous system of bolstering up quacks and quackery, to which so many of his cloth are prone.

We would respectfully suggest the propriety of the different Christian churches of the land taking order on this subject, and of their laying down the forms of dealing, as the Friends call it, with delinquents, whose conduct perils the dignity, purity, and usefulness of the different religious denominations to which they severally belong. The task would not be at all a difficult one, as, happily, the great body of the clergy, and especially the members of it most conspicuous for their piety, learning, and consistent walk, are, we believe, free from the taint of corruption in this matter; and they cannot but feel aggrieved at the sore not being cleansed.

Certificate-mongers, as well as their friends the nostrum-mongers, will be heard to say, that the medicines lauded by them have cured disease in very many cases. Discounting at a heavy rate the numbers claimed to have been relieved in this way, the remainder furnish no apology, still less justification, for quackery. The nostrums given are either inert or they possess undoubted activity. Under the first supposition, the cures which follow are Nature's cures, and so far no blame would seem to be attached to the nostrum. But there is this serious drawback to a reliance on such a means of medication: it lulls the fears of the sick and occupies their attention during the time when the disease (of the real nature of which they are ignorant) is making progress, and the opportune moment for suitable and active treatment is passed beyond recall. In epidemic, febrile, and some other diseases, the entire success of treatment, and consequently the life of the patient, mainly depend on the physician having an opportunity of prescribing within the first few hours, or at the furthest within the first day, after the attack. Under the other supposition, that the nostrum contains a substance of undoubted power, it is easy to see that, when taken by the patient at the particular stage of the disease in which it is indicated, it may exert a curative action; but as no rule or principle guides in its use, the chances of the appropriate time being lighted on are rare, and consequently the relief obtained must be equally rare, and always uncertain. But what can be anticipated from the effects of an active medicine when it is administered in all diseases and in every stage of disease, without regard to temperament, constitution, idiosyncrasy, or prior infirmities? What but aggravation of disease in the majority of cases in which it is thus indiscriminately administered, and death itself in many of these. Take quinine, or iodine, or opium, or strychnia, or digitalis, or mercury, or arsenic, or even aloes or jalap, as an example, and note down the results of its use when given in the same dose in every case of disease that offers. The very thought must make any considerate and humane man shudder, while he wonders at the perverted reasoning and blunted moral sense of those who prate about its being their duty, for the sake of humanity, to foster, by formal attestations, this wholesale and indiscriminate poisoning which is carried on inevitably by the sale and use of quack medicines. A prominent feature in the code of medical ethics is a strict if not stern prohibition against any physician giving his support, or even countenance, directly or indirectly, to quackery. This prohibition is based on the grounds just stated; and it is sustained and enforced as far as lie in their power by a vast majority of the members of the medical profession in all civilized and Christian countries. It is a part of their moral law, which experience shows to be alike necessary for the protection of the health of the people and for the usefulness and dignity of their own calling.

Happily for the interests of the community everywhere, sound views on this point are entertained by the leading religious journals, whose example ought to be more influential than it has been on others of minor note, which still persist in keeping up the nuisance of quack advertisements. Let us hope that they will all, seeing their error, come to the conclusion expressed in the following manly and benevolent declaration of the *English Evangelical Journal* for Jan. 1845 :—

"We shall cheerfully abandon, in future, the publication of all advertisements of quack medicines, which will be an act of homage to our own taste and judgment, no less than to the strongly-expressed opinion of some of our best friends, who, with ourselves, deeply deplore the disease and mortality occasioned by the nostrums of medical quacks, published daily in this great metropolis." We are glad to adduce at the same time the consistent and correct course, in this matter, of such an able and influential journal as the *New York Observer*, which will not allow of quackery being introduced, even by stealth, into its columns, under the garb of better company, without rebuke.

